# Chlorogenic acid ameliorates memory dysfunction via attenuating frontal lobe oxidative stress and apoptosis in diabetic rat model

**DOI:** 10.55730/1300-0144.5858

**Published:** 2023-12-18

**Authors:** Ramadhea Laila Afifa An-Nur Willya SAPUTRI, Nur ARFIAN, Junaedy YUNUS, Fauziyatul MUNAWAROH, Sagita Mega Sekar KENCANA, Dwi Cahyani Ratna SARI

**Affiliations:** 1 Department of Anatomy, Faculty of Medicine, Public Health and Nursing, Gadjah Mada University, Yogyakarta, Indonesia; 2 Master Program on Biomedical Sciences, Faculty of Medicine, Public Health and Nursing, Gadjah Mada University, Yogyakarta, Indonesia; 3 Faculty of Medicine, IPB University, Bogor, Indonesia

**Keywords:** Chlorogenic acid, Morris water maze, oxidative stress, apoptosis, diabetes

## Abstract

**Background/aim:**

Diabetes mellitus, characterized by hyperglycemia, causes various complications, one of which is memory dysfunction. The frontal lobe is known to be responsible for impaired memory function due to hyperglycemia and is associated with oxidative stress-mediated neuronal cell apoptosis. Chlorogenic acid (CGA) is reported to have neuroprotective effects. However, its effect on the frontal lobe in diabetes mellitus (DM) rats is not widely known. This research aimed to elucidate the effect of CGA on the mRNA expressions of SOD1, SOD2, p53, and Bcl-2 in the frontal lobe of DM rats.

**Materials and methods:**

Thirty male Wistar rats (2-month-old, 150–200 gBW) were randomly divided into six groups: C (control), DM1.5 (1.5-month DM), DM2 (2-month DM), CGA12.5, CGA25 and CGA50 (DM+CGA 12.5, 25, and 50 mg/kgBW, respectively). A single dose of streptozotocin (60 mg/kgBW) was intraperitoneally injected. Intraperitoneal CGA injection was administered daily for DM1.5 rats for 14 days. Path length was measured in the Morris water maze (MWM) probe test. After termination, the frontal lobes were carefully harvested for RNA extraction. Reverse transcriptase PCR was performed to examine the mRNA expression of SOD1, SOD2, p53, and Bcl-2.

**Results:**

The DM2 group demonstrated significant shorter path length on the MWM probe test and significantly lower mRNA expression of SOD1 and Bcl-2, compared to the C group. After CGA administration, the CGA25 group showed a significantly shorter path length than the C group. The CGA12.5 and CGA25 groups had significantly higher mRNA expression of SOD1 than the DM1.5 group. Compared to the DM1.5 and DM2 groups, SOD2 mRNA expression of the administration of all three CGA doses increased markedly. Furthermore, Bcl-2 mRNA expression was significantly increased in the CGA12.5 and CGA50 groups, compared with the DM2 group.

**Conclusion:**

Chlorogenic acid might improve memory function through upregulation of frontal lobes’ SOD1, SOD2, and Bcl-2 mRNA expression in DM rats.

## 1. Introduction

Diabetes mellitus (DM) is a metabolic disease that is commonly found in the population and is often associated with several complications, such as kidney injury, heart disease, and brain abnormalities [1,2]. Previous studies reported that brain injury in DM patients is shown by micro- and macrostructure changes of the brain. These changes manifest as progressive cognitive deterioration, cerebral infarction, cerebral atrophy, and neurodegeneration [3]. Perantie et al. [4] and Yonguc et al. [5] showed that the brain, specifically the hippocampus (region CA1 and CA3) and frontal cortex, underwent changes and contributed to acquisition and retention memory function impairment after hyperglycemic conditions. The retention memory function in rodents can be assessed using the Morris water maze (MWM) probe test [6].

Oxidative stress has been widely accepted to be a key mediator of the development of diabetes and its complications, due to increased free radicals and the failure of antioxidant defenses [2,5–6]. Hyperglycemia causes tissue and endothelial damage through the polyol pathway, formation of advance glycation end-products (AGE), activation of protein kinase C (PKC), and hexosamine pathway which further generate reactive oxidative species (ROS) [9]. Under hyperglycemic conditions, the antioxidant superoxide dismutase (SOD) enzyme is unable to eliminate the accumulation of ROS [10]. ROS play an important role in the pathogenesis of diabetic complications, depending on its effect on the target tissues [9]. The target cells, including the glomerular mesangial cells, the capillary endothelial cells of the retina, and the neuronal cells, are incapable of adequately regulating the concentrations of intracellular glucose in the diabetic ambience [9]. Excessive quantities of ROS oxidize various biomolecules like DNA, proteins, carbohydrates, and lipids, producing oxidative stress [9]. DNA damage due to stress condition can activate the p53 protein, which is a regulator of the proapoptosis Bax protein because it is reported that there is a p53-binding site on the Bax gene promoter [11]. As a response to cell death, p53 protein also rapidly translocates to mitochondria and physically interacts with the antiapoptosis Bcl-2 protein [10–11]. Therefore, p53 plays a role in the regulation of cell fate through the Bax/Bcl-2 ratio [13]. Memory dysfunction due to hyperglycemia is often associated with oxidative stress-mediated neuronal cell apoptosis [7].

Chlorogenic acid (CGA) can be found in green coffee and tea extracts and contains phenolic acids which are known to play roles as antioxidant, antiinflammatory, and central nervous system stimulators [14]. An in vivo study using rats reported that CGA could improve diabetic nephropathy by reducing oxidative stress and the response to inflammation in the kidney [15]. The neuroprotective effect of CGA was found to improve memory function of the rat model of transient global ischemia through upregulation of antioxidant and antiapoptotic compounds [16]. However, the effect of CGA on memory dysfunction due to progressive DM is still unknown. This study aimed to examine the effect of CGA administration on the mRNA expression of SOD1, SOD2, p53, and Bcl-2 in the frontal lobe of rats with DM.

## 2. Materials and methods

### 2.1. Experimental designs

Thirty male Wistar rats (aged 2 months, weighing 150–200 g) were housed in cages in the Anatomy Laboratory of the Faculty of Medicine, UGM, Yogyakarta. The environment was maintained with a light-dark cycle of 12:12 h, a room temperature of 26–31 °C, and a humidity level of 70%–90%. Rats were placed in separate cages between groups and fed standard AIN-93A and drinking boiled water ad libitum. This study has received approval from the Ethics Committee of the UGM FK-KMK on June 7th, 2022, with the approval number KE/FK/0700/EC/2022.

Rats were randomly divided into six groups, namely, group C (control), DM1.5 (1.5-month DM), DM2 (2-month DM), CGA12.5, CGA25, and CGA50 (DM+CGA dose 12.5, 25, and 50 mg/kgBW, respectively). A single dose of streptozotocin (STZ) (Cayman Chemical, Item No. 13104) at 60 mg/kgBW was administered via intraperitoneal injection to induce DM [17]. Intraperitoneal CGA (Sigma-Aldrich, USA, Cat. #3878-1G) injection was administered at 1.5 months after DM induction, daily for 14 days. Intraperitoneal saline injection for group C was administered in equivalent amounts. Glucose level was measured with a portable glucometer from the tail vein. A blood glucose level greater than 250 mg/dL is considered indicative of diabetes. The Morris water maze test was conducted before termination to assess the improvement of memory function.

### 2.2. Morris water maze (MWM) test

The Morris water maze (MWM) test consists of a circular tank (1.8 m in diameter and 0.5 m high) filled with water (20–24 °C). A platform (13 cm in diameter and 16.5 cm high) is placed 1.5–2.5 cm below the water surface, providing an escape for the test subjects. The surface of the water is made opaque using milk. The water tank is divided into 4 virtual quadrants. The platform was placed in the same position for each rat in each experiment. MWM was performed for 6 consecutive days by each rat. For the first 5 days, the rats were allowed to swim until they finally found and climbed the platform. On day 6, the platform was retrieved, and a probe test was performed to assess the spatial memory retention ability [6]. The probe test was carried out for 120 s. The path length in the target quadrant was recorded using a video camera placed above the tank and measured using a computer.

### 2.3. Termination and tissue preparation

Rats from groups C and DM1.5 were terminated at one-and-a-half months after the establishment of the animal model, while rats from groups DM2, CGA12.5, CGA25, and CGA50 were terminated at two months. Termination was performed via intraperitoneal injection of ketamine at a dose of 100 mg/kgBW. Next, an incision was made from the abdominal region to the thorax until the heart was exposed. Perfusion was performed using a 0.9% NaCl solution through the left ventricle of the heart until the blood was completely flushed out. The calvaria cranii of the rats were then opened. The cerebral hemispheres were separated, the meninges were dissected open, and then the anterior third was excised to isolate the frontal lobe. The left frontal lobes were placed in RNAlater stabilization solution (Ambion, AM7021). The right frontal lobes were fixed in 4% paraformaldehyde in PBS for 24 h and then embedded in paraffin for subsequent immunohistochemical staining examination.

### 2.4. RNA extraction and reverse transcriptase-PCR (RT-PCR)

The frontal lobes were extracted using GENEzol RNA solution (GENEzol, GZR100) according to the manufacturer’s protocol. The RNA concentration was calculated using a NanoDrop spectrophotometer (Maestrogen, MN-913A). RNA was synthesized into cDNA using a cDNA synthesis kit (SMOBio, RP1400) under PCR conditions of 30 °C for 10 min, 42 °C for 60 min, and 85 °C for 5 min. Subsequently, the cDNA was stored in a refrigerator at −20 °C. Reverse transcriptase-PCR was performed to amplify specific target genes, namely, SOD1, SOD2, p53, Bcl-2, and β-actin with the primer sequences as shown in Table 1.

The cDNA was mixed with primers and Taq Master Mix (GoTaq Green, M7122), followed by incubation at 94 °C denaturation for 10 s, annealing at 55 °C for SOD1, p53, and Bcl-2, at 58 °C for SOD2, and at 54 °C for β-actin for 30 s, an extension phase at 72 °C for 1 min, and a final extension phase at 72 °C for 10 min for 35 cycles. The PCR products were separated using 2% agarose gel with 100 bp DNA ladder (SMOBio, DM2400). Gene expressions were quantified with densitometry analysis using ImageJ software. β-actin mRNA expression was used as the housekeeping gene.

### 2.5. Immunohistochemical (IHC) staining

Immunohistochemical staining was done using p53 antibody (p53 Rabbit pAb, ABclonal A5761, 400X dilution) after heated in citrate buffer and inhibited for endogenous peroxide using 3% H_2_O_2_ in PBS. The antibodies were incubated overnight, followed by a 1-h incubation with the appropriate secondary antibody. Positive p53 IHC staining appeared as brown coloration within the nuclei of frontal lobe cells when observed under the microscope at magnifications of 100× and 400×.

### 2.6. Statistical analysis

The obtained data were analyzed using SPSS 25.0 software (IBM Corp., Armonk, NY, USA). A normality test using the Shapiro–Wilk method was conducted to determine the distribution of the data. Data that were normally distributed (p > 0.05) were subjected to a one-way ANOVA test, followed by a post hoc Fisher’s LSD test. Data that were not normally distributed (p < 0.05) were subjected to the Kruskal–Wallis H test, followed by a post hoc test using the Kruskal–Wallis one-way ANOVA. The data is statistically significant if the probability value of p < 0.05. Simple regression test and the Spearman test were employed to asses for correlation. A p-value of less than 0.05 was considered statistically significant.

## 3. Results

### 3.1. CGA affected blood glucose level

**Figure 1:**
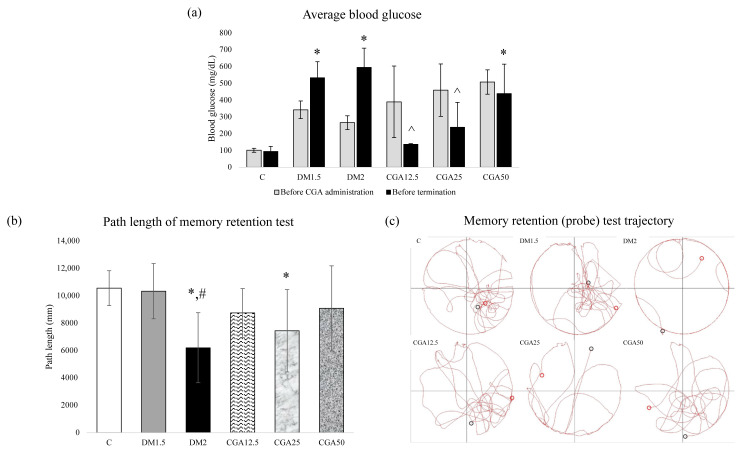
(a) Average blood glucose level before CGA administration vs. before termination, (b) CGA effects on path length of memory retention test and (c) their probe test trajectory. Data are represented as mean + SD. (*p < 0.05 vs C; #p < 0.05 vs DM1.5; ^p < 0.05 vs DM2).

Blood glucose was measured before termination. The comparison of blood glucose means + SD between the 6 groups is shown in Figure 1a [18]. The mean + SD of blood glucose in all groups, except the control group, were greater than 250 mg/dL. We demonstrated that streptozotocin injection resulted in higher blood glucose compared to the control group (p < 0.05). According to Furman, if the blood glucose levels of rats given STZ are greater than 250 mg/dL and/or statistically significantly higher than those of the control group, it indicates that the experimental animal model exhibits hyperglycemia and can be utilized for further studies on the DM model [17]. After chlorogenic acid administration, the blood glucose levels of CGA50 group were higher compared to those of the control group. However, blood glucose levels significantly decreased after CGA doses of 12.5 and 25 mg/kgBW compared to the DM2 group (p < 0.05). Nevertheless, there were no differences between the CGA-treated groups.

### 3.2. CGA might restore spatial memory deficit

Total path length in millimeters was measured while the rats were in the target quadrant to examine the memory retention. The comparison of the path length of the memory retention test means + SD between the 6 groups is shown in Figure 1b [18]. The path length of the DM1.5 group did not exhibit any differences from that of the C group. However, the DM2 group showed a statistically significantly shorter path compared to group C (p = 0.003) and group DM1.5 (p = 0.02). After chlorogenic acid administration, the CGA25 group showed a statistically significantly shorter path compared to the C group (p = 0.036). Although not statistically significant, the path lengths in the target quadrant of the CGA12.5 and CGA50 groups were longer than those of the DM2 group but were not different than those of the C group. There was no difference between the three doses of chlorogenic acid administration.

The trajectory of the probe test for the DM rats is depicted in Figure 1c [18]. The target quadrant was located in the right-bottom quadrant. From the figure, it is observed that the control and DM1.5 groups concentrated on the target quadrant, whereas the DM2 groups did not concentrate in the target quadrant. Following chlorogenic acid administration, the CGA12.5 and CGA50 groups concentrated in the target quadrant, while the CGA25 group did not concentrate in the target quadrant.

### 3.3. CGA elevated antiapoptosis Bcl-2 expression

**Figure 2:**
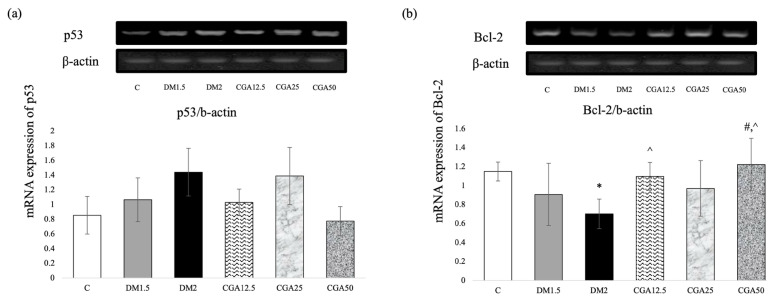
CGA effects in the frontal lobe of DM rats of (a) proapoptosis p53 and (b) antiapoptosis Bcl-2 mRNA expressions. Data are represented as mean + SD. (*p < 0.05 vs C; #p < 0.05 vs DM1.5; ^p < 0.05 vs DM2).

The mRNA expression of p53/β-actin means + SD between the six groups is shown in Figure 2a [18]. The Kruskal–Wallis H test demonstrated that there was no significant difference between the groups in the proapoptosis p53 mRNA expression.

The mRNA expression of Bcl-2/β-actin means + SD among the six groups is shown in Figure 2b [18]. The Bcl-2 mRNA expression in the DM1.5 and DM2 groups were lower than that in the C group, but only the DM2 group showed a statistically significant difference (p = 0.019). In comparison to the DM2 group, CGA administration showed that the Bcl-2 mRNA expressions were higher in the CGA12.5 (p = 0.043) and CGA50 (p = 0.005) groups, which was statistically significant. Furthermore, the Bcl-2 mRNA expression of the CGA50 group was significantly higher than in the DM1.5 group (p = 0.034).

### 3.4. CGA increased antioxidant superoxide dismutase enzymes

**Figure 3:**
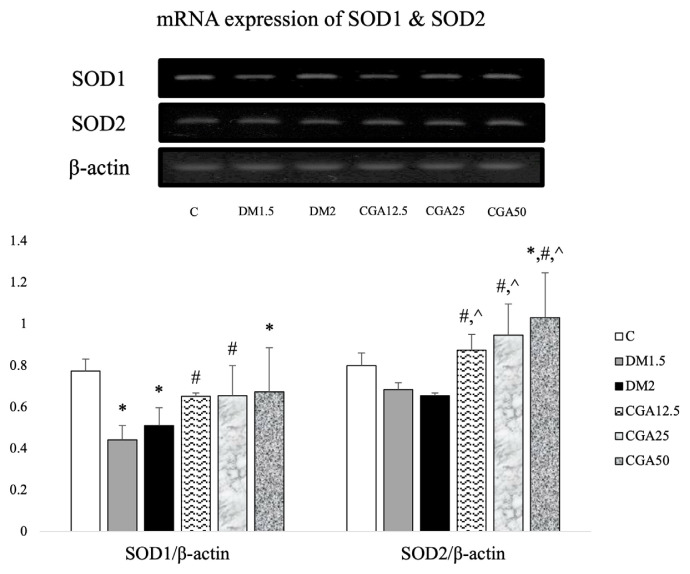
CGA effects in the frontal lobe of DM rats of SOD1 & SOD2 mRNA expressions. Data are represented as mean + SD. (*p < 0.05 vs C; #p < 0.05 vs DM1.5; ^p < 0.05 vs DM2).

The mRNA expression of SOD1/β-actin and SOD2/β-actin means + SD among the six groups is shown in Figure 3 [18]. We did not observe any difference in SOD2 mRNA expression in the DM1.5 and DM2 groups compared to the control group. However, there was a significantly lowered SOD1 mRNA expression in the DM1.5 (p = 0.002) and DM2 (p = 0.037) groups compared to the C group. CGA treatment significantly increased SOD1 mRNA expression in the CGA12.5 (p = 0.024) and CGA25 (p = 0.016) groups compared to the DM1.5 group. Furthermore, the mRNA expression of SOD1 in the CGA50 group was lower than that in group C, which was statistically significant (p = 0.038). In addition to that, administration of CGA significantly elevated SOD2 mRNA expression in the CGA12.5 (p = 0.023), CGA25 (p = 0.008), and CGA50 (p = 0.003) groups compared to the DM1.5 group, as well as the CGA12.5 (p = 0.013), CGA25 (p = 0.004), and CGA50 (p = 0.001) groups compared to the DM2 group. Additionally, the CGA50 group also showed significantly higher SOD2 mRNA expression compared to the C group (p = 0.039). There was no difference between the three doses of chlorogenic acid administration on mRNA expression of SOD1 and SOD2.

### 3.5. Amelioration of retention memory dysfunction statistically correlated with elevation of antioxidant SOD1 enzyme

The probe test path length was statistically correlated with the SOD1/β-actin mRNA expression (r = 0.414, p = 0.029, [n= 28]). However, a weak correlation was detected between the probe test path length and both SOD2/β-actin mRNA expression and blood glucose (r = 0.179, p = 0.381, [n= 26] and (r = −0.205, p = 0.276, [n= 30], respectively) (Table 2) [18].

## 4. Discussion

This study examined the effect of various doses of CGA on the expression of oxidative stress and apoptosis genes in the frontal lobe of DM rats. Before termination, the blood glucose of each rat was measured. Blood glucose levels in rats given STZ were greater than 250 mg/dL and/or statistically significantly higher than those in the control group. This indicates that the experimental animal model exhibited hyperglycemia and could be utilized for further studies on the DM model [17]. The administration of CGA at doses of 12.5 and 25 mg/kgBW in DM rats resulted in lower blood glucose level than those in the DM2 group. According to Miao and Xiang [19], CGA can delay glucose absorption in the intestine by inhibiting glucose-6-phosphate transferase I enzyme and lowering apical glucose transport driven by natrium gradient [20]. In vitro and in vivo studies also revealed that CGA lowered glucose export from the liver by inhibiting glucose-6-phosphatase [20–21].

This study revealed that CGA ameliorated memory function performance in diabetic rats. Diabetic rats exhibited memory dysfunction with an egocentric pattern and a reduction in path length during the probe test as shown in Figures 1b and 1c [18]. These findings indicate a dysfunction in retention memory. The frontal lobe plays a role in retention memory function [23] and is a part of the brain that is sensitive to glycemic control [4]. Memory function can be determined by the preference of rodents in the platform area when the platform is not available, also known as the probe test [6]. If the animal can remember the location of the platform during the recognition test, it will swim a longer path in the target quadrant [24]. This study aligns with previous research indicating that animal models of type 1 DM, which involve the prefrontal cortex [6,24] and hippocampus [2], experience impaired spatial learning and memory function loss.

Chlorogenic acid can restore spatial memory deficit conditions [14,25]. In a recent study, CGA administration showed that DM rats tended to have good memory function. This was reflected by the path length of the CGA12.5 and CGA50 groups being longer than that of the DM2 group, but not significantly different from the C group. However, a previous study reported that CGA-treated groups (12.5, 25, and 50 mg/kgBW) had longer time in the target quadrant of the MWM probe test compared to the DM rats group [27]. The CGA12.5 group also performed better in the escape latency test compared to the DM rats [28]. Other studies reported that CGA can prevent diabetes-induced learning and memory impairment [29]. Our study revealed that hypoglycemic condition and memory improvement based on MWM test (total path length) may be correlated with each other; however, the effect of CGA on memory improvement correlates with its hypoglycemic effect. Future research with anti-hyperglycemic substance may be needed for elucidating these results.

The crucial pathogenic factor associated with diabetes-associated cognitive decline (DACD) is apoptosis signaling [2]. In a recent study, there was no significant difference between groups in p53 mRNA expression in the frontal lobe of DM rats. However, the Bcl-2 mRNA expression of DM rats was significantly lower compared to the control rats [18]. A previous study reported higher mRNA expression of p53 in DM rats’ hippocampus, which was localized by positive p53 immunoreactivity on hippocampus’ pyramidal cells [28]. This study is consistent with the previous study in that after exposure to stress, neuronal culture cells underwent apoptosis, indicated by a shrinking nucleus and decreased expression of Bcl-2 [30]. DNA damage response due to diabetes activates the p53 protein that leads to the induction of cell cycle arrest, DNA repair, cell senescence, autophagy, and apoptosis [31]. Activated p53 induces apoptosis both via extrinsic and intrinsic pathways [32]. In the extrinsic pathway, p53 induces expressions of death receptor on cell surface; thus, the cell undergoes apoptosis [33]. In the intrinsic pathway, apoptosis is mediated by mitochondria, where activated p53 induces the release of mitochondrial apoptosis factors such as Bax, NOXA, PUMA, and cytochrome C into the cytosol. These factors then form the apoptosome, which triggers caspase-9 activation, initiating apoptosis execution [8,31]. The B-cell lymphoma 2 (Bcl-2) family is a key regulator of apoptosis because it bridges extrinsic and intrinsic pathways [35]. The Bcl-xL and Bcl-2 proteins play a role in antiapoptosis, inhibiting Bax and BAK proteins [35].

After chlorogenic acid administration, this study demonstrated a tendency to reduced p53 mRNA expression in the CGA12.5 and CGA50 groups than in the DM2 group [18]. Chlorogenic acid administration returned the condition as shown by lower proapoptosis p53 and Bax mRNA expression [28]. The CGA dose of 12.5 and 50 mg/kgBW in DM rats showed significantly higher antiapoptotic Bcl-2 mRNA expression compared to the 2-month DM group [18]. This recent study corroborates previous findings suggesting that chlorogenic acid has a neuroprotective effect by increasing antiapoptotic proteins Bcl-2 and Bcl-xL. It achieves this by upregulating antiapoptotic proteins like Bcl-2 and Bcl-xL, while downregulating caspase-3 cleavage in H_2_O_2_-induced neuronal cells [36]. In vivo studies have also shown that chlorogenic acid can protect hippocampal pyramidal cells by increasing the mRNA expression of Bcl-2 [16].

Much evidence suggests that apoptosis in many diseases may be triggered by heightened oxidative stress and inflammatory reactions [2]. In a recent study, impaired memory function was followed by decreased mRNA expression of SOD1 in the frontal lobe of DM rats compared to the control [18]. Oxidative stress has been proven to play a role in cognitive decline related to hyperglycemia [37]. Streptozotocin-induced diabetes animal models are widely used to study oxidative stress as a pathogenesis of memory impairment due to diabetes [24,36]. Many studies have reported that the concentration of ROS is significantly increased in the brain of DM rats [39]. However, under hyperglycemic conditions, the antioxidant SOD enzyme is unable to eliminate ROS [10]. Another previous study also stated that STZ-induced mice showed a decreased activity, mRNA, and protein expression of antioxidant SOD1 and catalase enzymes [40]. Oxidative stress in cells can affect the translocation of transcription factors to the nucleus [41]. Therefore, a decrease in the antioxidant enzyme in diabetes could occur due to oxidation of transcription factors that initiated the transcription process [40].

High blood glucose interferes with electron transport in mitochondria, which will result in increased oxygen oxidation by coenzyme Q and form superoxide [42]. Manganese superoxide dismutase (Mn-SOD/SOD2) is an antioxidant enzyme that is responsible for mitochondrial detoxification against oxygen radicals by catalyzing the dismutation of superoxide free radicals [43]. Hyperglycemic conditions can cause ROS overproduction which further reduces the antioxidant capacity of cells [42]. In this study, there was no difference of SOD2 mRNA expression in the frontal lobe between the DM and control rats. There is a dynamic regulation of antioxidant enzymes to maintain the redox balance [43]. It is caused by ROS, as second messengers, which act as second messengers and trigger various activities against radicals that can harm cells under basal conditions [44]. An in vitro study reported that the decrease in SOD1 activity was due to increased SOD2 expression [43]. Another study reported decreased total SOD and SOD1 activity but increased SOD2 activity in the heart of DM mice [45]. Furthermore, in early diabetes, tissues increase the activity of antioxidant enzymes to defend against hyperglycemia-induced ROS production [45].

Chlorogenic acid can increase the expression of antioxidant enzymes by donating its hydrogen atoms to free radicals [16]. In this study, administration of CGA doses of 12.5 and 25 mg/kgBW showed significantly higher SOD1 mRNA expression compared to DM rats without CGA. In addition, the administration of CGA showed significantly higher SOD2 mRNA expression than the DM rats. In the present study, the longer path in the target area by the DM rats with CGA also statistically correlated with higher antioxidant SOD1 mRNA expression, but weakly correlated with lower blood glucose (Table 2) [18]. A previous study reported that CGA, as an antioxidant, acts by chelating cations [46]. CGA chelated the copper ion of SOD1 and the manganese ion of SOD2 and deposited them on the liver of obese Zucker rats [46]. Phenol compounds in CGA can also stimulate nuclear factor-erythroid 2-related factor 2 (Nrf2), a transcription factor that regulates genes encoding various antioxidant enzymes, to bind to antioxidant response elements (ARE) [19].

The DM1.5 group was formed to observe any progression of complications due to DM in the frontal lobe of rats. Memory function in 1.5 months after STZ administration had not shown any disturbance compared to the control. There was also no significant difference in the mRNA expression of the observed genes compared to the control. The pathogenesis from hyperglycemia in diabetic encephalopathy, which is characterized by changes in cognitive function and nervous system structure, is a long process accompanied by other metabolic abnormalities [47]. Five weeks after STZ administration, no clear cognitive deficits were observed, but an increased rate of apoptosis in the hippocampus was evident in rats, as confirmed by the TUNEL assay [47]. Administration of STZ for studying diabetic complications could be repeated at the seventh week [17], and the study could then continue for several more weeks or months.[17].

This study used several doses of intraperitoneal CGA to determine the relationship between dose and its effect on the frontal lobe of DM rats. However, no differences were found between the three doses in terms of memory function and the examined genes of oxidative stress and apoptosis. This study also demonstrated inconsistency in the CGA administration at a dose of 25 and 50 mg/kgBW. Chlorogenic acid is not well absorbed by the gastrointestinal tract when given orally and its concentrations are more stable when administered intraperitoneally [48]. Previous studies reported that chlorogenic acid at a dose of 30 mg/kgBW had a positive effect on brain damage due to focal [26] and global [16] cerebral ischemia. High doses of CGA have been reported to induce 4/6 rat death, while low doses were considered nontoxic [49].

Another possible mechanism that may affect brain injury and cognitive dysfunction associated with hyperglycemia is vasculopathy or angiopathy [7]. The animal model of diabetes stroke showed endothelial cell dysfunction and increased vascular permeability [50]. Interestingly, CGA could prevent spatial memory deterioration and affect the vascular response by decreasing the expression of vasoconstrictor ET-1 and increasing the expression of endothelial cell CD31 in global brain ischemic rats [16]. Further research investigating the effect of chlorogenic acid on the vascular response in memory dysfunction due to diabetes mellitus would be worthwhile.

## 5. Conclusion

Chlorogenic acid (CGA) treatment in the frontal lobe of DM rats might improve memory function by increasing its antioxidant and antiapoptosis properties. Further basic-to-clinical research are considered because CGA has antioxidant and antiapoptosis effect that will likely result in clinical benefit as a supplementary therapy in treating or preventing memory dysfunction related to diabetic condition.

## Figures and Tables

**Table 1:** Primer sequences for reverse-transcriptase PCR.

Gene	Primer sequence
SOD1	Forward: 5’-GCGGTGAACCAGTTGTGGTG-3’ Reverse: 3’-AGCCACATTGCCCAGGTCTC-5’
SOD2	Forward: 5’-ATGTTGTGTCGGGCGGCGTGCAGC-3’ Reverse: 3’-GCGCCTCGTGGTACTTCTCCTCGGTG-5’
p53	Forward: 5’-CCCCTGAAGACTGGATAACTGT-3’ Reverse: 3’-ATTAGGTGACCCTGTCGCTG-5’
Bcl-2	Forward: 5’-GCGTCAACAGGGAGATGTCA-3’ Reverse: 3’-TTCCACAAAGGCATCCCAGC-5’
b-actin	Forward: 5’-TTCCACAAAGGCATCCCAGC-3’ Reverse: 3’-TTCCACAAAGGCATCCCAGC-5’

**Table 2:** Statistical analysis of correlation between probe test path length, blood glucose, and antioxidant superoxide enzyme.

		Blood glucose	SOD1/β-actin	SOD2/β-actin
Path length	Correlation coefficient	−0.205	0.414	0.179
	Sig. (2-tailed)	0.276	0.029	0.381
	N	30	28	26
